# Previous Experience Seems Crucial to Eliminate the Sex Gap in Geometry Learning When Solving a Navigation Task in Rats (*Rattus norvegicus*)

**DOI:** 10.3389/fpsyg.2022.838407

**Published:** 2022-05-09

**Authors:** Alejandra Aguilar-Latorre, Víctor Romera-Nicolás, Elisabet Gimeno, V. D. Chamizo

**Affiliations:** ^1^Departament de Cognició, Desenvolupament i Psicologia de l’Educació, Universitat de Barcelona, Barcelona, Spain; ^2^Institut de Neurociències, Universitat de Barcelona, Barcelona, Spain

**Keywords:** geometry learning, landmark learning, sex differences, Morris pool, rats

## Abstract

There is much evidence, both in humans and rodents, that while navigating males tend to use geometric information whereas females rely more on landmarks. The present work attempts to alter the geometry bias in female rats. In Experiment 1 three groups of female rats were trained in a triangular-shaped pool to find a hidden platform, whose location was defined in terms of two sources of information, a landmark outside the pool and a particular corner of the pool. On a subsequent test trial with the triangular pool and no landmark, females with prior experience with two other pool shapes–with a kite-shaped pool and with a rectangular-shaped pool (Group Long Previous Experience, LPE), were significantly more accurate than control rats without such prior experience (Group No Previous Experience, NPE). Rats with a short previous experience–with the rectangular-shaped pool only (Group Short Previous Experience, SPE) did not differ from Group NPE. These results suggest that the previous experience with different shaped-pools could counteract the geometry bias in female rats. Then, Experiment 2A directly compared the performance of LPE males and females of Experiment 1, although conducting several test trials (i.e., shape, landmark, and preference). The differences between males and females disappeared in the three tests. Moreover, in a final test trial both males and females could identify the correct corner in an incomplete pool by its local, instead of global, properties. Finally, Experiment 2B compared the performance of NPE rats, males and females, of Experiment 1. On the test trial with the triangular pool and no landmark, males were significantly more accurate than females. The results are explained in the framework of selective attention.

## Introduction

It has been claimed that animals can encode geometric information spontaneously, even when trained in the presence of salient visual cues. Cheng (1986) carried out pioneering work with male rats in a rectangular arena. In this apparatus, three walls were black (the two short walls and one of the long walls), while the other long wall was white. At each of the four corners of the arena there were also other distinctive visual and olfactory cues (i.e., non-geometric cues). The food was hidden in one of the four corners of the arena. The rats quickly located the food in the correct corner, but simultaneously also made rotational errors, as they also looked for the food in the corner diagonally opposite the correct one (a corner geometrically identical to the correct one–i.e., long wall to the right and short wall to the left). These results indicate that the geometry of the apparatus guided animal behaviour, despite the fact that the other visual and olfactory cues (i.e., non-geometric cues) better predicted the position of the hidden food. This kind of “primacy” of geometric information makes ecological sense, since the shape of many environments remains unchanged throughout the year, which does not happen with non-geometric information such as vegetation (for reviews see Tommasi et al., 2012; McGregor, 2017). Could it be core or innate knowledge? (Spelke and Kinzler, 2007). According to Spelke and Kinzler (2007), various studies carried out with human babies and also with non-human animals have shown that there are four main knowledge systems, and that one of them captures the geometry of the environment (such as distances, angles, and sense relationships between extended surfaces).

However, some studies show results that are difficult to explain according to the previous statements, as male and female rats behave differently when it comes to geometry issues (Williams et al., 1990; Golob and Taube, 2002; Rodríguez et al., 2010; Keeley et al., 2013; Torres et al., 2014). The same is true with human participants (Galea and Kimura, 1993; Dabbs et al., 1998; Saucier et al., 2002; Choi and Silverman, 2003). Both male rats and men seem to perform best when using Euclidean information (like distances and directions), while female rats and women seem to perform best when using landmark information. Even studies conducted with fish, a class of vertebrates phylogenetically distant from mammals and humans, suggest a sex difference in rewarded geometric and featural tasks (Sovrano et al., 2003; Lee et al., 2012).

Williams et al. (1990) carried out a highly influential study with male and female rats in a radial maze. The rats were trained to asymptotic performance and then various manipulations, test trials, to the geometry of the room or to the landmarks, were carried out. The performance of the males was not altered by changes in the landmarks as long as the geometry of the room did not change; but if the geometry of the room was changed, the performance was greatly affected, even if the landmarks were present. Very different was what happened with the females, whose performance was greatly affected by the rearrangement of the landmarks (and this was the case with changes or without changes in the geometry of the room); however, the females performed well in the absence of the landmarks as long as the geometry of the room was not altered. A subsequent study by Rodríguez et al. (2010), with male and female rats in a Morris pool, has revealed similar effects. These results suggest that geometry is more salient for males and landmarks more salient for females. A further study by Rodríguez et al. (2011b), where cue competition designs were used, confirmed this claim. Employing a similar apparatus as Rodríguez et al. (2010), the study by Keeley et al. (2013) found a significant correlation between all measures of the water maze performance and entorhinal cortex (EC) volume only in males–the EC, as well as the hippocampus, are crucial in the codification of position and direction to a specific goal during navigation. Furthermore, when cyclicity was taken into account, these authors found no differences between females.

In the study by Golob and Taube (2002), male and female rats were trained in a white rectangular arena in two different tasks: one aversive and one appetitive. The aversive task was conducted in a modified Morris pool in which, to escape from the water, the rats had to swim to the corner where the platform was (i.e., a wet condition); in the appetitive task, the rats were thirsty and they were reinforced with water when they approached the correct corner (i.e., a dry condition). In the two conditions a salient landmark was always present to disambiguate the correct corner and the rats had to perform a spatial delayed match-to-sample task. Specifically, they were given blocks of two trials (i.e., a sample trial which was always followed by a test trial). In the sample trial, the rats had to search for the correct corner, the one that contained a reinforcer (either the escape platform, in the wet condition; or water, in the dry condition). Then, in the test trial, the animals were reinforced if they approached the corner previously reinforced in the sample trial. The reinforced corner varied randomly across the different blocks of trials. In the wet condition, the authors could not replicate Cheng’s (1986) results: rotation errors when looking at the corner geometrically identical to the correct one, so frequent in Cheng’s work, were not found in females. According to Golob and Taube (2002) it is important to differentiate between appetitive and aversive tasks. Replicating Cheng’s (1986) results would be expected in the appetitive tasks but not in the aversive tasks, such as a Morris pool.

However, a recent set of experiments by Chamizo et al. (2020), using male and female rats and several modified Morris pools (as in the wet condition of Golob and Taube’s, 2002), has shown that both male and female rats primarily encode geometric information even when trained in the presence of salient visual cues, thus supporting Cheng’s initial results (Cheng, 1986; see also Gallistel, 1990). In Experiments 1 and 2 of the study by Chamizo et al. (2020), male and female rats were trained in a modified pool (i.e., with a rectangular shape) to find a hidden platform, which was located based on two cues or sources of information, both next to the platform: one specific landmark (a target landmark), outside the pool, and one particular corner of the pool (a target corner). In both experiments the results revealed that geometry learning had clearly interfered with learning about the target landmark. Rats’ learning about the target landmark was negligible. These findings were expected in male rats (Rodríguez et al., 2010, 2011b; Keeley et al., 2013) but totally unexpected in females. Then in Experiment 3 (Chamizo et al., 2020) the authors addressed the concept of task difficulty. The following speculation was carried out. If learning in the rectangular-shaped pool was easier than learning in the triangular-shaped pool used by Rodríguez et al. (2010, Experiment 2; see also Chamizo et al., 2016; Rodríguez et al., 2011b; Keeley et al., 2013), in part because the swimming surface was half the size in the rectangular pool than in the triangular pool, the results of Experiments 1 and 2 could be explained by appealing to the proposal of Coluccia and Louse (2004) that emphasises that sex differences tend to appear only when the task to be learned is difficult (see Forcano et al., 2009, for a demonstration in a Morris pool). Experiment 3 (Chamizo et al., 2020) was carried out in order to see if there would be differences in the speed with which the male and female rats learned a spatial task, a simple one, that was based solely on geometry. The experiment consisted of two groups of male rats and two groups of female rats and two shaped-pools (a rectangular shape and a triangular shape–as in Rodríguez et al., 2010) were used. The results revealed that groups rectangle reached the platform faster than groups triangle, thus showing a different level of difficulty in the two shaped pools (for the same result in a preliminary study, see Rodríguez and Chamizo, 2013). Moreover, Experiment 3 clearly showed that males performed better than females only in the more difficult task (i.e., in the triangular pool); male and female rats did not differ in the easier task (i.e., in the rectangular pool). In conclusion, male and female rats learned just as quickly when training was carried out in the rectangular pool, but male rats learned faster than females when they were trained in the pool with a triangular shape. The experiment supports the proposal of Coluccia and Louse (2004), which claim that sex differences tend to appear only when the task to be learned is difficult, thus offering an explanation for the previous results of Experiment 1 and Experiment 2.

But many questions are still unanswered, waiting for further research. For example, could the previous sex differences in the triangular pool (Keeley et al., 2013; Rodríguez et al., 2010; Chamizo et al., 2016, 2020) disappear with prior experience with geometry? The main aim of the present work is to answer this question. The study consists of two experiments, both with different pool-shapes and inspired in the Rodríguez et al. (2010) work. It is important to mention that in the study by Rodríguez et al. (2010, Experiment 1) it was examined whether the estrous cycle of female rats influenced their performance. It was found a lack of differences within females when cyclicity was taken into account (and for the same results see Keeley et al., 2013; with a circular pool see Rodríguez et al., 2011a). Following these null results, in the present work the rats’ estrus cycle was not measured to avoid causing them an unnecessary stress.

## Experiment 1

In the study by Rodríguez et al. (2010, Experiment 2), male and female rats were trained in a triangular-shaped pool to find a hidden platform, whose location was defined in terms of two sources of information: a landmark outside the pool and a particular corner of the pool with a distinctive shape. After training, three test trials without the platform were conducted: a preference test (i.e., a conflict test) and two learning tests (i.e., a landmark test and a shape test). On any test trial there were two recording areas and the time the subjects spent in these two areas was measured. The preference test pitted the two sources of information against one another and a clear sex difference was found: males spent more time in the distinctive corner of the pool, while females spent more time in an area of the pool next to the landmark. The learning tests revealed that both males and females had learned about the two cues: landmark and geometry. Moreover, a clear male advantage on shape learning was also found. A question worth answering is the following. Would it be possible to improve the female rats’ performance in the shape test of the Rodríguez et al. (2010, Experiment 2) protocol by means of prior experience with different pool-shapes? The aim of Experiment 1, a preliminary experiment, was to answer this question. The design of the experiment is illustrated in Table 1.

**TABLE 1:** Design of Experiment 1 (*n* = 36♀–12 per group).

Groups	Training 1 *(white and blue curtains)*	Shape test 1 *(white and blue curtains)*	Training 2 *(white and blue curtains)*	Shape test 2 *(white and blue curtains)*	Training 3 *(black curtains)*	Shape test 3 *(black curtains)*
LPE	Kite → ptf	Kite	Rectangle → ptf	Rectangle	Triangle + X → ptf	Triangle (without X)
	
	Preex. Kite		Preex. Rectangle			

SPE			Rectangle → ptf	Rectangle	Triangle + X → ptf	Triangle (without X)
	
			Preex. Rectangle			

NPE					Triangle + X → ptf	Triangle (without X)

*Preex., Preexposure; Kite, kite-shaped pool; Rectangle, rectangular-shaped pool; Triangle, unusual triangular-shaped pool; X, landmark; ptf, platform. Note that in Training 1 and Training 2, the LPE and SPE groups have Learning rats and Preexposure rats.*

The experiment was conducted with three groups of female rats: Group Long Previous Experience (LPE), Group Short Previous Experience (SPE), and Group No Previous Experience—rats with no previous experience (NPE). LPE rats were first trained in a kite-shaped pool (Training 1); for half the rats, they had to find a submerged platform located in a fixed position in a specific corner of the pool, whereas for the remainder of the rats they were pre-exposed to the kite-shaped pool. This phase was followed by a second acquisition phase in a rectangular-shaped pool (Training 2); for half the rats, they had to find a submerged platform located in a fixed position in a specific corner of the pool, whereas for the remainder of the rats they were pre-exposed to the rectangular pool. SPE rats were trained in the rectangular-shaped pool only (Training 2); for half the rats, they had to find a submerged platform located in a fixed position in a specific corner of the pool, whereas for the remainder of the rats they were pre-exposed to the rectangular pool. Group NPE, control rats, did not receive any training in either the kite-shaped pool or the rectangular-shaped pool. The two subgroups (i.e., learning rats and preexposure rats), of Group LPE and Group SPE were used to compare these two procedures, shape learning and shape preexposure, in female rats. Then, all rats (LPE, SPE, NPE) were trained in a triangular-shaped pool (as in Rodríguez et al., 2010, Experiment 2) to find a hidden platform, whose location was defined in terms of two sources of information: one landmark next to the platform, but outside the pool, and one particular corner of the pool (Training 3). Following Training 3, the rats received a single test trial with the triangular pool and no landmark (i.e., a shape test). On this test the main prediction was: Group LPE > Group SPE > Group NPE. Would that be the case?

### Methods

#### Subjects

The subjects were 36 naive females, Long Evans rats, from our own colony, approximately 3 months old at the beginning of the experiment. They were divided into three groups, with twelve females each group. The animals were housed in standard cages, 25 × 15 × 50 cm, in groups of two and were maintained on ad lib food and water, in a colony room with a 12:12-h light-dark cycle. They underwent the experiment within the first 8 h of the light cycle. The three main groups were named LPE, SPE, NPE, with two of them, LPE and SPE, being initially subdivided into two subgroups (Learning and Preexposure–*n* = 6 each of them). They were all matched for latency to find the platform on pretraining trials.

#### Apparatus

**FIGURE 1:**
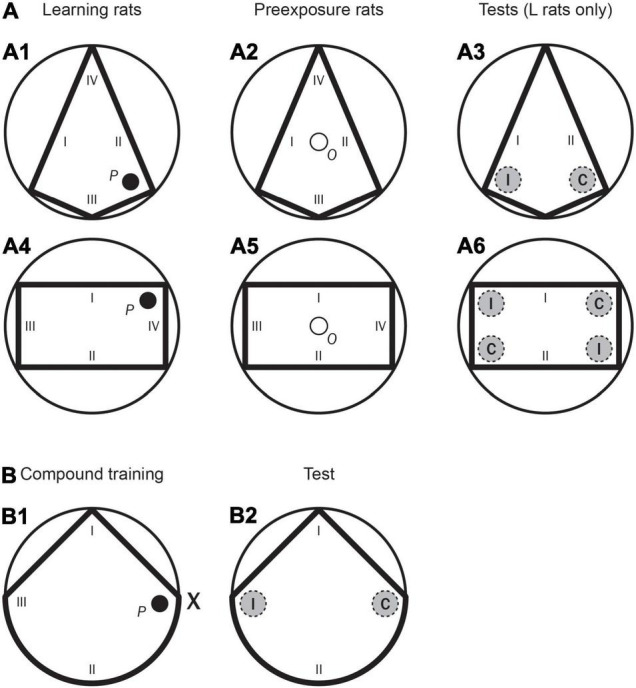
Experiment 1. (**A:** A1) A schematic representation of the kite shaped pool, as well as the position of the hidden platform (*P*) and the starting positions (I–IV) used in the first training phase for Groups LPE and SPE (Learning rats). (A2) A schematic representation of the kite shaped pool as well as the position of the observation compartment (*O*), and the starting positions (I–IV) used in the first training phase for Groups LPE and SPE (Preexposure rats). (A3) A schematic representation of the kite shaped pool, as well as the two recording areas (correct and incorrect, C and I, respectively) and the starting positions (I, II) used in the test trial for rats in Groups LPE and SPE. (A4) A schematic representation of the rectangular pool as well as the position of the hidden platform (*P*), and the starting positions (I–IV) used in the second training phase for Groups LPE and SPE (Learning rats). (A5) A schematic representation of the rectangular pool as well as the position of the observation compartment (*O*), and the starting positions (I–IV) used in the second training phase for Groups LPE and SPE (Preexposure rats). (A6) A schematic representation of the rectangular pool, as well as the four recording areas (two correct and two incorrect, C and I, respectively) and the starting positions (I, II) used in the test trial for rats in Groups LPE and SPE. (**B:** B1) A schematic representation of the triangular pool and the position of the landmark, X, as well as the position of the hidden platform (*P*), and the starting positions (I–III) used in the compound training phase for all rats. (B2) A schematic representation of the triangular pool, as well as the two recording areas (correct and incorrect, C and I, respectively) and the starting positions (I, II) used in the test trial for all rats.

The apparatus was a circular swimming pool made of plastic and fibreglass and modelled after that used by Morris (1981). It measured 1.58-m in diameter and 0.40-m deep, and it was filled to a depth of 0.30-m with water rendered opaque by the addition of 1 cl/l of latex. The water temperature was maintained at 22 + 1°C. The pool was situated in the middle of a large room and mounted on a wooden platform 0.43-m above the floor. To create the different pool shapes (i.e., kite, rectangle, and unusual triangle) several acrylic boards were inserted in the pool resting on platforms at the base, which supported them vertically. For the kite shape, four boards were used. The short ones were 39.5 cm high, 0.5 cm thick, and 58 cm long. The resting two boards, the large ones, were 39.5 cm high, 0.5 cm thick, and 145 cm long. For the Learning rats (groups LPE and SPE) a platform, *P*, was placed in the kite shape right corner of the pool, with a straight short wall to the right and a straight long wall to the left–as shown in Figure 1A1. For the Preexposure rats (groups LPE and SPE), an observation compartment, *O*, was placed in the middle of the kite shape–as shown in Figure 1A2. To create the rectangular shape, four boards were used. The short ones were 39.5 cm high, 0.5 cm thick, and 58 cm long. The resting two boards, the large ones, were 39.5 cm high, 0.5 cm thick, and 145 cm long. For the Learning rats (groups LPE and SPE), a platform, *P*, was placed in the rectangular shape upper right corner of the pool, with a straight short wall to the right and a straight long wall to the left–as shown in Figure 1A4. For the Preexposure rats (groups LPE and SPE), the observation compartment, *O*, was mounted on a base that was placed in the middle of the rectangular shape–as shown in Figure 1A5. To create the triangular shape two boards were used forming an angle of 90°. The boards were 39.5 cm high, 0.5 cm thick, and 112 cm long. In the three pool shapes, the height of the top of the boards coincided with the pool border. The pools were surrounded by curtains (either plain black or white colour with horizontal blue stripes) reaching from ceiling to the base of the pool, forming a circular enclosure 2.4 m in diameter. In the triangular shape, inside the black enclosure, above the border of the pool, a single object could be placed. It was suspended from a false ceiling, 35 cm above the surface of the water. The object, landmark X, was a skittle (6 cm in diameter at the base and 16.5 cm in height, with the wider part measuring 26 cm in circumference, with blue and yellow segments). For all rats (groups LPE, SPE, and NPE) this single landmark as well as the point formed by a straight wall to the left and a circular wall to the right of the two added boards, could define the location of the platform–as shown in Figure 1B1. In the three pool shapes (kite shape, rectangular shape, and triangular shape), when a platform, *P*, was used it was a circular platform 11 cm in diameter and made of transparent Perspex, mounted on a base, with its top 1 cm below the surface of the water; when an observation compartment, *O*, was used (kite shape and rectangular shape only) it was a circular plate, placed in the middle of the pool, 22 cm in diameter and made of white earthenware mounted on a base, with its top 1 cm above the surface of the water. In the triangular shape, the platform was placed 38 cm from the point formed by the corner of the pool with a straight wall to the left, and the circular base of the triangle to the right, on a line that bisected the centre of the pool. In the kite shape and rectangular shape, the platform was placed 8 cm from the target corner. In order to ensure that the rats used these sources of information (the shape of the pool in the kite shape and rectangular shape pools; and the landmark and the shape of the pool in the triangular shape pool) to locate the platform, rather than any inadvertently remaining static room cues (like noises from pipes and air conditioning), the boards of the pool shapes and the platform (as well as the landmark in the triangular shape) were semi-randomly rotated with respect to the room (90°, 180°, 270°, or 360°) with the restriction that all four positions of the room were used each day. For the three pool shapes, a closed-circuit video camera with a wide-angle lens was mounted 1.75-m above the centre of the pool inside a false ceiling, and its picture was relayed to recording equipment in an adjacent room.

#### Procedure

Because Experiment 1, and also Experiments 2A and 2B, is based on the work of Rodríguez et al. (2010, Experiment 2), in those phases that reproduce this protocol (pretraining, and Training 3 in Tables 1, 2) the background curtains were black (as in the aforementioned work). However, in the new phases for animals to have experience with different pool-shapes (Training 1 and Training 2 in Tables 1, 2) the colour of the background curtains was white with horizontal blue stripes (instead of plain black). The aim of this manipulation was to “surprise” the animals in the third training phase, with the change of the curtains’ colour (a similar manipulation, although for other purposes, was used by Mesa et al., 2017, Experiment 3) thus reducing the possibility of a blocking effect from previous geometries (i.e., in other words, to avoid that shape learning could block or reduce landmark learning in the third training phase, Training 3).

**TABLE 2:** Procedure of Experiment 1 (*n* = 36♀–12 per group).

Training phase	Procedure	Groups				
		LPE (*n* = 12♀)		SPE (*n* = 12♀)		NPE (*n* = 12♀)
		Learning (*n* = 6♀)	Prexposure (*n* = 6♀)	Learning (*n* = 6♀)	Prexposure (*n* = 6♀)	
Training 1: Kite-shaped pool	Training	• 5 days (8 trials/day). • Rats had 120 s to find the platform (*P* in Figure 1A1). • The time to reach the platform was measured.	• 5 days (8 trials/day). • On each trial rats were placed in an observation compartment (*O* in Figure 1A2) for 60 s.			
	Test day	• 8 training trials. • 1 test trial of 60 s without the platform (kite shape test). • Two recording areas (C and I in Figure 1A3). • The time spent in the recording areas was measured.				
Training 2: Rectangular-shaped pool	Training	• 5 days (8 trials/day). • Rats had 120 s to find the platform (*P* in Figure 1A4). • The time to reach the platform was measured.	• 5 days (8 trials/day). • On each trial rats were placed in an observation compartment (*O* in Figure 1A5) for 60 s.	• 5 days (8 trials/day). • Rats had 120 s to find the platform (*P* in Figure 1A4). • The time to reach the platform was measured.	• 5 days (8 trials/day). • On each trial rats were placed in an observation compartment (*O* in Figure 1A5) for 60 s.	
	Test day	• 8 training trials. • 1 test trial of 60 s without the platform. • Four recording areas (C and I in Figure 1A6). • The time spent in the recording areas was measured.		• 8 training trials. • 1 test trial of 60 s without the platform. • Four recording areas (C and I in Figure 1A6). • The time spent in the recording areas was measured.		

Training 3 (for all groups): Triangular-shaped pool + landmark	Training	• 5 days (8 trials/day). • Rats had 120 s to find the platform (*P* in Figure 1B1). • The time to reach the platform was measured.				
	Test day	• 8 training trials. • 1 test trial of 60 s without the platform. • Only the triangular-shaped pool was present, the landmark was removed (see Figure 1B2). • Two recording areas (C and I in Figure 1B2). • The time spent in the recording areas was measured.				

There were five types of trial: pretraining, shape learning, shape preexposure, shape and landmark learning, and test. A procedure was employed in the pre-exposure trials that permitted good spatial learning when the rats were simply allowed to observe the relevant geometry-it was similar to that used by Rodrigo et al. (1997) and Prados et al. (1999). Pretraining, shape learning, and shape and landmark learning were escape trials (i.e., the rats learned to escape from the water by swimming directly to the platform from different points of the pool). Pretraining consisted of placing a rat into the circular pool, which was surrounded by back curtains, without the boards or landmark, but with the hidden platform present. The rat was given 120 s to find the platform, and once the rat had found it, it was allowed to stay on it for 30 s. If it had not found the platform within the 120 s, it was picked up, placed on it, and left there for 30 s. The platform was moved from one trial to the next, and the rat was placed in the pool in a different location on each trial, as far as possible equally often on the same or opposite side of the pool from the platform, and with the platform to the right or to the left of where the rat was placed. Rats were given five such pretraining trials over 2 days, with two trials on Day 1, and three on Day 2.

The first training phase (i.e., Training 1 in Tables 1, 2) was conducted with the white and blue curtains in the kite shaped pool (as shown in Figure 1A1). Only Group LPE had this phase. The procedure for the Learning rats was similar to that of pretraining. Rats were given eight trials per day over 5 days in the kite shaped pool (a total of 40 trials). These trials had an ITI (i.e., an inter trial interval) of 8–10 min, and the platform and boards were rotated between trials. The procedure for the Preexposure rats took place in the observation compartment, which was always situated in the centre of the pool (as shown in Figure 1A2). A trial of preexposure consisted of placing a rat in this compartment and leaving it there for 1 min. Rats were given eight trials of preexposure per day over 5 days in the kite shaped pool (a total of 40 trials). These trials had an ITI of 8–10 min, and the boards were rotated between trials.

The second training phase (i.e., Training 2 in Tables 1, 2) was conducted with the white and blue curtains in the rectangular pool (as shown in Figure 1A4). Only two groups, Group LPE and Group SPE, had this phase. The procedure for the Learning rats was similar to that of pretraining. Rats were given eight trials per day over 5 days in the rectangular shaped pool (a total of 40 trials). These trials had an ITI of 8–10 min, and the platform and boards were rotated between trials. The procedure for the Preexposure rats took place in the observation compartment (as shown in Figure 1A5). Rats were given eight trials of preexposure per day over 5 days in the rectangular pool (a total of 40 trials). These trials had an ITI of 8–10 min, and the boards were rotated between trials.

For Learning rats, after both the first and the second training phases (in Group LPE), and after the second training phase only (in Group SPE), there was a test day with eight training trials (identical to the previous training phase), followed by one test trial (in the kite shaped pool after the first training phase, and in the rectangular pool after the second training phase) without the platform (see Tables 1, 2). Test trials were always 60 s long. On a test trial (here and also in Experiments 2A and 2B) there were two recording areas (each of them 22 cm in diameter–twice the hidden platform diameter). In the kite shaped pool, the amount of time the rat spent in the two different areas, one in front of the correct corner (C, in Figure 1A3) and one in exactly the opposite corner (incorrect corner, I, in the previous figure), was recorded. Each rat was placed in the pool from one specific position (at I and II only, as shown in Figure 1A3). In the rectangular pool, the amount of time the rat spent in two different areas (i.e., correct and incorrect − C and I, respectively) was recorded–as shown in Figure 1A6. The correct area was defined as having a short wall to the right and a long wall to the left. Two of the four corners correspond to this description (they are geometrically identical). These two corners were considered the “correct area” (C). The remaining two corners (also geometrically identical) were defined as having a long wall to the right and a short wall to the left. These two corners were considered the “incorrect area” (I). Rats were placed in the pool individually from one specific position (at I and II only, as shown in Figure 1A6).

The third training phase (i.e., Training 3 in Tables 1, 2) was conducted with the black curtains (as in pretraining) in the unusual triangular pool, and with the landmark, X, always present–as shown in the Figure 1B1. All rats (Groups LPE, SPE, and NPE) had this phase. The procedure for the shape and landmark learning trials was similar to that of pretraining. The rat was placed in the pool in a different location on each trial, as far as possible equally often with the platform to the right, to the left or in front of where the rat was placed (at I, II, and III of the previous figure). Rats were given eight trials per day over 5 days (a total of 40 trials). These trials had an ITI of 8–10 min, and the platform, landmark, and triangular shape were rotated between trials.

After the third training phase, there was a test day with eight training trials (identical to the training phase), followed by one test trial (in the triangular pool and in the absence of the landmark, as shown in the Figure 1B2) without the platform. On the test trial the amount of time the rat spent in two different areas, one in front of the correct corner (C), and one in front of the incorrect corner (I) was recorded. The rats were placed in the pool from one specific position (at I and II only, as shown in the previous figure). These starting positions were randomly determined. Throughout the entire experiment, rats were run in groups of eight and spent the intertrial interval (ITI) in small individual compartments.

#### Data Analysis

In Experiment 1 (as well as in Experiments 2A and 2B), the data were analysed using two-samples and paired t-tests as well as analysis of variance (ANOVA) on SPSS (IBM Corp, 2017). Significant interactions were analysed through simple main effects and *post hoc* pairwise comparisons. An alpha level of 0.05 was adopted for all the statistical analyses.

### Results and Discussion

We conducted preliminary analyses (both in the escape latencies, on days 17 and 18, and in the time spent in the correct and the incorrect areas on the test trial, on day 18) with the 24 rats with previous experience (Learning and Preexposure) to see if these animals differed. On the ANOVAs of the escape data of days 17 and 18 the variables taken into account were Experience (Learning and Preexposure) and Group (LPE and SPE); on the ANOVA of the test trial with geometry (in the triangular pool) the variables taken into account were Experience (Learning and Preexposure), Group (LPE and SPE), and Area (correct and incorrect). As no significant differences were found in any case among rats with previous experience (Learning and Preexposure), in the remaining analysis of Experiment 1 reported here, LPE rats (50% of which have a learning experience and the other 50% a pre-exposure experience) and SPE animals (50% of which have a learning experience and the other 50% a pre-exposure experience) will be considered as two main groups: LPE and SPE (i.e., Long Previous Experience and Short Previous Experience, respectively).

**FIGURE 2:**
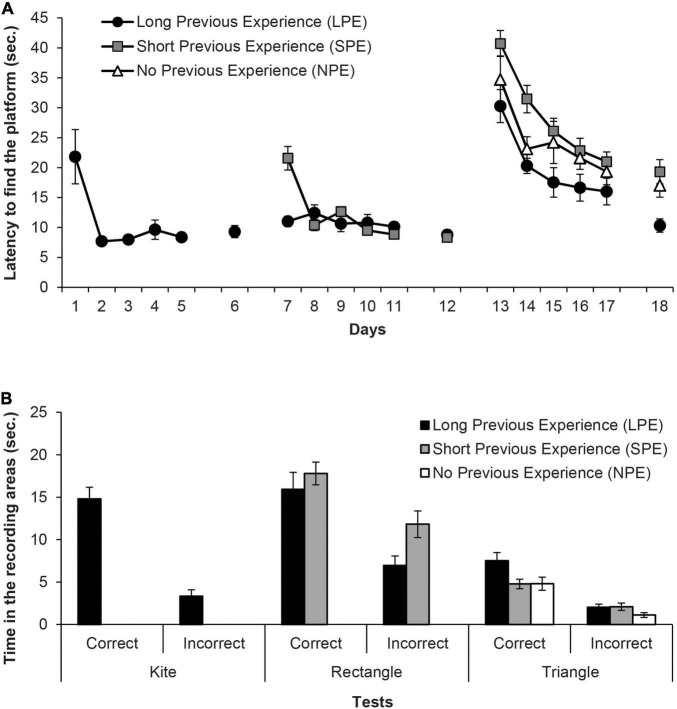
**(A)** Mean escape latencies by the subjects in Experiment 1 during the three training phases and the escape trials of the test days. Error bars denote standard error of the mean. **(B)** Mean time spent by the subjects in Experiment 1 in the two recording areas (correct and incorrect) during the Kite test trial (Group LPE), Rectangle test trial (Groups LPE and SPE) and in the final Triangle test trial (Groups LPE, SPE, and NPE). Error bars denote standard error of means.

Figure 2A shows the mean escape latencies of rats during all the experiment (Days 1–18). Four independent ANOVAs and one *t*-test were conducted to analyse these latencies. The repeated measures ANOVA of the first training phase (Days 1–5, LPE group only) showed that assumption of sphericity had been violated, Mauchly’s test χ^2^(9) = 36.15, *p* < 0.001, therefore degrees of freedom were corrected using Greenhouse-Geisser estimate of sphericity (ε = 0.32). The ANOVA showed that the variable Days was significant, *F*(1.29,6.44) = 7.97, *p* = 0.024, η^2^_p_ = 0.62, and rats reached the platform faster as days progressed. In the second training phase, Group LPS continued to perform accurately, while Group NPS rapidly reached the platform faster as days progressed. The mixed ANOVA of the escape latencies during the second training phase (Days 7–11, LPE and SPE groups only), taking into account the variables Group (LPE and SPE) and Days, revealed that the variable Days was significant, *F*(4,40) = 13.86, *p* < 0.001, η^2^_p_ = 0.58, as well as the Days × Group interaction, *F*(4,40) = 13.43, *p* < 0.001, η^2^_p_ = 0.57, while the main effect of Group was non-significant, *F*(1,10) = 2.93, *p* = 0.118. Only on day 7, LPE rats reached the platform faster than SPE animals (*p* = 0.001). The independent two-samples *t*-test for Day 12 (LPE and SPE groups only) showed no significant differences in the escape latencies of both groups, *t*(10) = 0.46, *p* = 0.653.

In the third training phase, all groups showed very high latencies at the beginning of this phase. As the only group without prior experience with pool-geometry (except on pre-training with the circular pool) is Group NPE, this result indicates that the “extra” surprise due to the change of the curtains’ colour in Group LPE and Group NPE has been effective. The mixed ANOVA of the third training phase (Days 13–17, all groups), taking into account the variables Group (LPE, SPE and NPE) and Days, did not meet the assumption of sphericity, χ^2^(9) = 35.67, *p* < 0.001, thus degrees of freedom of the within-subject factors were corrected using Greenhouse-Geisser estimate (ε = 0.68). The ANOVA revealed that both Days and Group variables were significant, *F*(2.7,132) = 27.28, *p* < 0.001, η^2^_p_ = 0.45 and *F*(2,33) = 8.19, *p* = 0.001, η^2^_p_ = 0.33, respectively. *Post hoc* pairwise comparisons using Bonferroni correction showed that Group LPE reached the platform faster than Group SPE (*p* = 0.001), but did not significantly differ from Group NPE (*p* = 0.111); additionally, Group SPE and Group NPE did not differ between the two (*p* = 0.210). The Days × Group interaction was non-significant, *F*(5.4,89.16) = 0.7, *p* = 0.694. Finally, the one-way ANOVA of the escape trials on Day 18 showed that the variable Group was significant, *F*(2,33) = 7.08, *p* = 0.003, η^2^_p_ = 0.30. Subsequent pairwise comparisons using Bonferroni correction revealed that Group LPE reached the platform faster than both Group SPE and Group NPE, *p* = 0.003 and *p* = 0.033, respectively, which did not significantly differ from each other (*p* > 0.99).

Figure 2B shows the time spent in the correct and the incorrect areas by the groups during the 60 s of each test (i.e., in the kite shaped pool for Group LPE only, in the rectangular pool for Group LPS and Group SPE, and in the triangular pool for all groups). The paired *t*-test for the test trial with the kite shaped pool on Group LPE revealed that rats spent more time in the correct area than in the incorrect area, *t*(5) = 8.47, *p* < 0.001, *d* = 3.45. The implication is that the rats had learned about the correct corner. The mixed ANOVA on the data of the test trial with the rectangular pool, considering the variables Group (LPE and SPE) and Area (correct and incorrect), showed that the variable Area was significant, *F*(1,10) = 42.15, *p* < 0.001, η^2^_p_ = 0.81. No other main effect or interaction was significant (*F*s ≤ 3.32). All rats spent more time in the correct area than in the incorrect area, which implies that they had learned about the correct corner. Finally, the mixed ANOVA on the data of the test trial with the triangular pool revealed that the variable Area was significant, *F*(1,33) = 54.96, *p* < 0.001, η^2^_p_ = 0.63, as well as the variable Group, *F*(2,33) = 5.64, *p* = 0.008, η^2^_p_ = 0.26, although the Area × Group interaction was non-significant *F*(2,33) = 2.35, *p* = 0.111. All rats spent more time in the correct area than in the incorrect area, which implies that they had learned about the correct corner. And most important, *post hoc* pairwise comparisons using Bonferroni correction showed that Group LPE had a better performance than Group NPE (*p* = 0.008), and was close to differ from Group SPE (*p* = 0.067), while groups SPE and NPE did not differ between them (*p* > 0.99). These last results seem to show that a long prior experience with different pool-geometries could alter the female rats’ learning in the shape test of the Rodríguez et al. (2010) protocol.

All rats improved their performance as days went by in the three training phases and the change of the curtains’ colour in the third training phase seems to have been effective (for a good example of an attenuation of blocking when a change of context is introduced between the two phases of the blocking design see Weaver and Gordon, 1988–and for a demonstration of an unblocking effect in a Morris pool due to a change in the platform position between the two phases of training see Rodrigo et al., 2005). This is especially evident when observing the high latencies of the LPE and SPE rats on the first day of this phase. On the test trial with the triangular pool and no landmark, Group LPR performed better than the other two groups (Group SPE and Group NPE), which did not differ between them.

In Experiment 1 it was found that prior unreinforced exposure to multiple shaped-pools (i.e., preexposure animals in Group LPE) facilitated the learning of the platform position when it was subsequently introduced during Training 3. This result seems compatible both with latent learning and with perceptual learning. Early experiments on latent learning found that unreinforced exposure to a multiple-unit maze will facilitate the learning of the true path when reward is subsequently introduced. And pioneering experiments on perceptual learning (Gibson and Walk, 1956; Channell and Hall, 1981; Chamizo and Mackintosh, 1989) have found that prior exposure to complex stimuli may also facilitate their subsequent discrimination (for a whole issue on recent advances in perceptual learning see Delamater et al., 2021). The results of the learning animals of the same group, Group LPE, are compatible with selective attention (Mackintosh, 1975). Mackintosh (1975) proposes changes in the associability of, or attention to, particular stimuli, dependent on their relative predictive value. Interestingly, the two subgroups of rats (i.e., Preexposure and Learning) ended up showing the same facilitated learning regarding the shape cue following compound training (i.e., Training 3). In conclusion, prior experience with different pool-shapes (i.e., Group LPE), although not after just one pool-shape (Group SPE), could improve the female rats’ performance in the shape test of the Rodríguez et al. (2010, Experiment 2) protocol.

## Experiment 2A

In Experiment 2A we asked whether prior learning with different pool-shapes will reduce the gap, or even eliminate it, between males and females on the test trial with the triangular pool and no landmark of the Rodríguez et al. (2010, Experiment 2) protocol (for other demonstrations of this result see also Rodríguez et al., 2011b,^2013^; Torres et al., 2014; Chamizo et al., 2016). An additional purpose of Experiment 2A was to provide some information about the remaining test trials, the landmark test and the preference test, of the mentioned protocol. A final aim of this experiment was to check if both males and females could identify the correct corner of a specific pool-shape by its local properties, instead of global properties, as Pearce et al. (2004–see also McGregor et al., 2006; for a review see Pearce, 2009) showed for the first time, but only with male rats. The design of the test trials of Experiment 2A is illustrated in Table 3.

**TABLE 3:** Design of Experiment 2A after Training 3 phase (i.e., tests trials–*n* = 8♂ and 8**♀**).

Groups	Shape test *(black curtains)*	Landmark test *(black curtains)*	Preference test *(black curtains)*	Retraining *(white and blue curtains)*	Global vs. Local test *(white and blue curtains)*
LPE♂ & ♀	Triangle (without X)	X (in circular pool)	Triangle + X (conflict test)	Kite + X	Incomplete kite (without X)

*Triangle, unusual triangular-shaped pool; X, landmark; Kite, kite-shaped pool; Incomplete kite, incomplete kite-shaped pool.*

Experiment 2A replicated the procedure used in the various phases of Experiment 1 for the Learning rats of Group LPE, although with a few exceptions. The first and most important refers to the test phase following the third training phase (i.e., Training 3 in Table 4). This phase was now followed by three test days (instead of one as in Experiment 1), and each day ended with a test trial (days 18–20): a preference or conflict test and two learning tests–a landmark test and a shape test (as in Rodríguez et al., 2010, Experiment 2). Secondly, following the three test days after the third training phase, all rats had one day of retraining with the kite-shaped pool followed by one test day (days 21 and 22–which were the same as in the first training phase). This final test day ended with a test trial with part of the kite-shaped pool only. The aim of this test was to check if the correct corner could be identified in the incomplete pool by its local properties (i.e., rather than by its position relative to the overall shape of the pool).

**TABLE 4:** Procedure of Experiment 2A (*n* = 8**♂** and 8♀) and 2B (*n* = 9♂ and 9♀).

Training phase	Procedure	Experiment	
		2A (LPE, ♂ & ♀ – *n* = 16)	2B (NPE, ♂ & ♀ – *n* = 18)
Training 1 (Exp. 2A only): Kite-shaped pool	Training	• 5 days (8 trials/day). • Rats had 120 s to find the platform (*P* in Figure 1A1). • The time to reach the platform was measured.	
	Test day	• 8 training trials. • 1 test trial of 60 s without the platform. • Two recording areas (C and I in Figure 1A3). • The time spent in the recording areas was measured.	
Training 2 (Exp. 2A only): Rectangular-shaped pool	Training	• 5 days (8 trials/day). • Rats had 120 s to find the platform (*P* in Figure 1A4). • The time to reach the platform was measured.	
	Test day	• 8 training trials. • 1 test trial of 60 s without the platform. • Four recording areas (C and I in Figure 1A6). • The time spent in the recording areas was measured.	

Training 3 (for both experiments): Triangular-shaped pool + landmark	Training	• 5 days (8 trials/day). • Rats had 120 s to find the platform (*P* in Figure 1B1). • The time to reach the platform was measured.	
	Test day (shape test)	• 8 training trials. • 1 test trial of 60 s without the platform. • Only the triangular-shaped pool was present, the landmark was removed (see Figure 3A1). • Two recording areas (C and I in Figure 3A1). • The time spent in the recording areas was measured.	
	Test day (landmark test)	• 8 training trials. • 1 test trial of 60 s without the platform. • Only the landmark was present, the triangular-shaped pool was removed (see Figure 3A2). • Two recording areas (C and I in Figure 3A2). • The time spent in the recording areas was measured.	
	Test day (preference test)	• 8 training trials. • 1 test trial of 60 s without the platform. • The landmark was placed in the opposite corner of the triangular-shaped pool (see Figure 3A3). • Two recording areas (L and S in Figure 3A3). • The time spent in the recording areas was measured.	

Retraining (Exp. 2A only): Kite-shaped pool	Retraining	• 8 training trials. • Rats had 120 s to find the platform (*P* in Figure 3B1). • The time to reach the platform was measured.	
	Test day (global vs. local test)	• 8 training trials. • 1 test trial of 60 s without the platform. • An incomplete kite shaped pool was used with only a short and a large board (see Figure 3B2). • Three recording areas (C, I_1_, and I_2_ in Figure 3B2). • The time spent in the recording areas was measured.	

Based on the results of Experiment 1, three predictions were hypothesised: (1) The first and most important, that the gap between males and females on the test trial with the triangular pool and no landmark firstly observed by Rodríguez et al. (2010) would disappear, (2) that the preference of females for the landmark cue firstly observed on the conflict test trial by Rodríguez et al. (2010) would disappear, (3) that both males and females could identify the correct corner in the incomplete pool by its local properties. Would that be the case?

### Methods

#### Subjects and Apparatus

**FIGURE 3:**
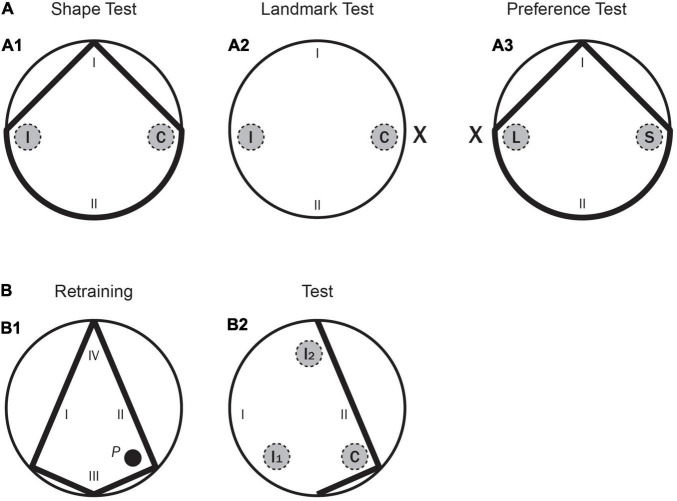
Experiment 2. **(A)** A schematic representation of the pool shapes, as well as the two recording areas (correct and incorrect, C and I, respectively, for the learning tests; landmark and shape, L and S, respectively, for the conflict test) and the starting positions (I, II) used in the main test trials. (A1) for the Shape test; (A2) for the Landmark test; (A3) for the Preference test. (**B:** B1) A schematic representation of the kite shaped pool, as well as the position of the hidden platform (*P*) and the starting positions (I–IV) used in the final Retraining phase. (B2) A schematic representation of part of the kite pool, as well as the three recording areas -correct, incorrect 1 and incorrect 2 (C, I1, and I2, respectively) and the starting positions (I–IV) used in the final test with the Incomplete Kite pool.

The subjects were naive Long Evans rats from our own colony: 8 males and 8 females, approximately 3 months old at the beginning of the experiment. They were divided into two groups, males and females. The animals were housed in standard cages, 25 × 15 × 50 cm, in same-sex groups of two. They were kept and maintained as in Experiment 1. The two groups were named Group LPE-Males and Group LPE-Females. The different shaped-pools, colour of the curtains in the different phases, and the experimental room were also the same as in Experiment 1, although with one exception. To create the incomplete kite shape needed for the final test trial only two boards were used, a short one and a large one (as shown in Figure 3B2). The hidden platform, *P*, and the geometry of the pool were situated as shown in the previous figure.

#### Procedure

As in Experiment 1, there were five types of trial: pretraining, shape learning, shape preexposure, shape and landmark learning, and test. The general procedures were exactly the same as those used in the previous experiment for the Learning rats of Group LPE, although with two exceptions. The first and most important is that in Experiment 2A the third training phase was followed by three test days, and each day ended with a test trial (as in Rodríguez et al., 2010, Experiment 2). The second refers to a final retraining day with the kite-shaped pool, followed by a test day which also ended with a test trial.

Rats were given five pretraining trials over 2 days, with two trials on Day 1, and three on Day 2. Training for the first and second training phases was conducted in the kite shaped pool and in the rectangular pool, respectively. Rats were given eight trials per day over 5 days in each pool (a total of 40 trials in each pool). These trials had an ITI of 8–10 min, and the platform and boards were rotated between trials. After both the first and the second training phases (i.e., Training 1 and Training 2 in Table 4), there was a test day with eight training trials (identical to the previous training phases), followed by one test trial (in the kite shaped pool after the first training phase, and in the rectangular pool after the second training phase) without the platform. Test trials were always 60 s long. All rats received a third training phase, as in Experiment 1. In this phase, a landmark, X, was always present, as well as two boards forming a triangular shape–as shown in Figure 1B1. Rats were given eight trials per day over 5 days (a total of 40 trials). These trials had an ITI of 8–10 min, and the platform, landmark, and triangular shape were rotated between trials. Following the third training phase, there were three consecutive test days, each starting with eight training trials (identical to Training 3), followed by one test trial without the platform. Test trials were always 60 s long and were counterbalanced over the 3 days. On one test trial the two sources of information, the landmark and the correct corner, were presented 180° apart–as shown in Figure 3A3. The amount of time the rat spent in two different areas. one in front of the landmark (L), and one in front of the correct corner (S), was recorded. The rats were placed in the pool from one specific position (at I and II only, as shown in the previous figure). These starting positions were randomly determined. In the other two test trials the rats were tested in the circular pool with the landmark (Figure 3A2) and in the triangular shaped pool with no landmark (Figure 3A1). The amount of time that the rats spent in the two different but identically sized areas (i.e., the target area, C, close to either the landmark or the previously correct corner and a control area 180° apart, I, as shown in Figures 3A1,A2) was recorded in each test. The reason for measuring the time spent in the control area as well as the target area was to check that on the shape test rats could discriminate between these two corners of the triangle, and on the landmark test to check whether they were simply swimming in circles at a certain distance from the wall of the pool.

Finally, all the rats had one day of re-training with the kite-shaped pool (which was exactly the same as in the first training phase, as shown in Figure 3B1) and then they received a final test trial with part of this pool only–as shown in Figure 3B2. The rats were placed in the incomplete kite-shaped pool from one specific position (at I and II only, as shown in the previous figure). The amount of time that the rats spent in three different but identically sized areas (i.e., the correct area, C, close to the previously correct corner and two incorrect areas, I1 and I2, as shown in Figure 3B2) was recorded in each test. Throughout the entire experiment, rats were run in groups of eight and spent the intertrial interval (ITI) in small individual compartments.

### Results and Discussion

**FIGURE 4:**
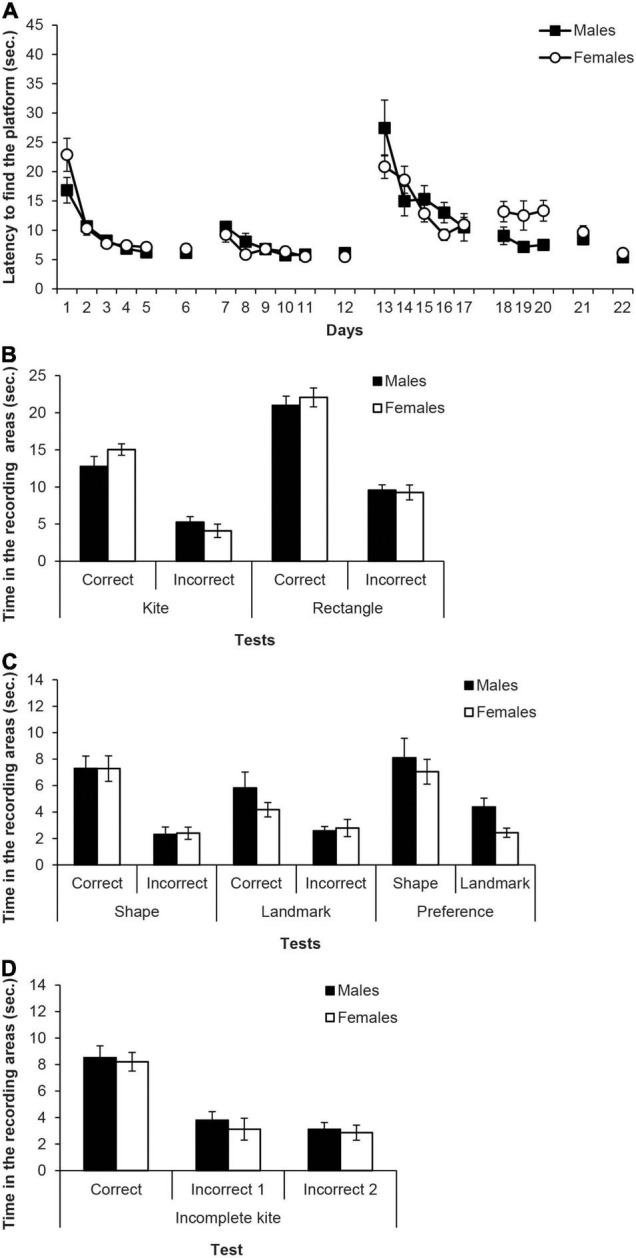
Experiment 2A. **(A)** Mean escape latencies by the subjects in Experiment 2 during the three training phases and the escape trials of the test days. Error bars denote standard error of the mean. **(B)** Mean time spent by the subjects in Experiment 2A in the two recording areas (correct and incorrect) during the Kite test trial and Rectangle test trial. **(C)** Mean time spent by the subjects in Experiment 2A in the two recording areas during the main test trials: Shape test and Landmark test (recording areas: correct and incorrect, C and I, respectively), and Preference test (recording areas: shape and landmark, S and L, respectively). Error bars denote standard error of means. **(D)** Mean time spent by the subjects in Experiment 2A in the three recording areas -correct, incorrect 1, and incorrect 2 (C, I1, and I2, respectively) during the final test trial with the Incomplete Kite. Error bars denote standard error of means.

Figure 4A shows the mean escape latencies of male and female rats during all the experiment (days 1–22). Four mixed ANOVAs and four independent two-samples *t*-tests were calculated to analyse these latencies with the within-subjects factor of Days and the between-groups factor of Sex (males, females). The mixed ANOVA of the first training phase with the kite-shaped pool (days 1–5) did not meet the assumption of sphericity, χ^2^(9) = 43.87, *p* < 0.001, thus degrees of freedom of the within-subject factors were corrected using Greenhouse-Geisser estimate (ε = 0.39). The ANOVA revealed that the only significant factor was Days, *F*(1.56,21.80) = 40.06, *p* < 0.001, η^2^_p_ = 0.74, and rats reached the platform faster as days progressed. No other main effect or interaction was significant (*F*s ≤ 4.46). The two-samples *t*-test on latencies of day 6 showed no significant differences between males and females *t*(14) = 0.72, *p* = 0.485. The mixed ANOVA of the second training phase with the rectangular pool using the Greenhouse-Geisser estimate [ε = 0.63, χ^2^(9) = 21.37, *p* < 0.012], revealed a significant effect of Days, *F*(2.55,35.74) = 12.24, *p* < 0.001, η^2^_p_ = 0.47, while no other main effect or interaction reached significance (*F*s ≤ 1.38). Again, rats reached the platform faster as days progressed. The two-samples *t*-test on day 12 did not find significant differences between the latencies of males and females *t*(14) = 0.88, *p* = 0.396. The mixed ANOVA of the third training phase with the triangular pool and the landmark (days 13–17) showed a significant effect of Days, *F*(4,56) = 20.26, *p* < 0.001, η^2^_p_ = 0.59, as well as the interaction Days × Sex, *F*(4,56) = 2.61, *p* = 0.045, η^2^_p_ = 0.16, while the main effect of Sex was not significant, *F*(1,14) = 0.47, *p* = 0.502. Here too, rats reached the platform faster as days progressed. As in Experiment 1, both groups showed very high latencies at the beginning of this phase. A result that clearly indicates that the “extra” surprise, due to the change of the curtains’ colour, has been effective. *Post hoc* pairwise comparisons using Bonferroni correction showed no significant sex differences across days (*p*s ≥ 0.78), but male rats escape latencies on days 14–17 were significantly shorter than those on day 13 (*p*s ≤ 0.014), while female rats reached the platform faster on days 16 and 17 compared to days 13 and 14 (*p*s ≤ 0.050). The mixed ANOVA of days 18–20 surprisingly found that the variable Sex was significant, *F*(1,14) = 8.11, *p* = 0.013, η^2^_p_ = 0.67, and males latencies were lower than those of females. No other factor or interactions were significant (*F*s ≤ 0.53). However, the *t*-test of the retraining day 21 latencies with the incomplete kite-shaped pool did not find any significant difference between male and female rats, *t*(14) = 0.90, *p* = 0.386. Finally, the *t*-test of day 22 revealed no significant sex difference on the latencies, *t*(14) = 1.74, *p* = 0.104.

Figure 4B shows the time spent in the recording areas (correct and incorrect) by the two sexes on the kite-shaped pool and the rectangular pool tests. For test trial with the kite-shaped pool, the mixed ANOVA taking into account Sex and Area, showed that the variable Area was significant, *F*(1,14) = 52.86, *p* < 0.001, η^2^_p_ = 0.79, and rats spent more time in the correct area than in the incorrect area. No other main effect or interaction was significant (*F*s ≤ 1.83). On the test trial with the rectangular pool, the mixed ANOVA revealed that the only significant effect was Area, *F*(1,14) = 99.97, *p* < 0.001, η^2^_p_ = 0.88, while other factors and interactions did not reach statistical significance (*F*s ≤ 0.33).

Figure 4C (left and middle) shows the time spent in the two recording areas on each learning test (i.e., shape and landmark). The mixed ANOVA of the two learning tests including the factors Area, Test and Sex found that the variable Area was significant, *F*(1,14) = 34.14, *p* < 0.001, η^2^_p_ = 0.73, as well as the interaction Test × Area, *F*(1,14) = 5.45, *p* = 0.035, η^2^_p_ = 0.28. Simple effect analyses showed that rats spent more time in the correct area than in the incorrect one, in both the shape and the landmark tests, *F*(1,14) = 26.62, *p* < 0.001, η^2^_p_ = 0.66 and *F*(1,14) = 13.28, *p* = 0.003, η^2^_p_ = 0.48, respectively. Moreover, rats spent more time in the correct area of the shape test than in the correct area of the landmark test, *F*(1,14) = 5.21, *p* = 0.039, η^2^_p_ = 0.27, while there was no significant difference between the tests in the incorrect area (*p* = 0.425). The main effect of Test was close to significance, *F*(1,14) = 3.56, *p* = 0.080, and no other factor or interaction was significant (*F*s ≤ 0.71). These results indicate that males and females performed equally well in the shape test and in the landmark test, and more importantly, that both sexes performed better in the shape than in the landmark test. Therefore, both sexes had learned more about the geometry cue than about the landmark cue.

Figure 4C (right) also shows the time in the shape and landmark areas on the preference test. The mixed ANOVA revealed that the effect of Area was significant, *F*(1,14) = 17.46, *p* = 0.001, η^2^_p_ = 0.56, and rats spent more time in the area controlled by the shape cue than in the one controlled by the landmark cue. No other main factor or interaction was significant (*F*s ≤ 2.74). This result reveals a clear preference for the geometry cue over the landmark cue both in male and female rats.

Figure 4D shows the time in the three recording areas on the incomplete kite-shaped pool. The mixed ANOVA found that the only significant variable was Area, *F*(1,14) = 27.45, *p* < 0.001, η^2^_p_ = 0.66. *Post hoc* pairwise comparisons with the Bonferroni correction revealed that rats spent more time in the correct area, C, than in the incorrect ones, I1 and I2, *p*s < 0.001, which did not significantly differ from each other.

In conclusion, all rats improved their performance as days went by in the three training phases and the change of context in the third training phase, the change of the curtains’ colour, seems to have been effective. This is especially evident when observing the abnormally high latencies of the rats on the first day of this phase. As predicted, the gap between males and females that was typically observed in the past in the shape or geometry test trial has disappeared, as well as the preference of females for the landmark cue. Finally, both males and females could identify the correct corner in the incomplete pool by its local properties.

## Experiment 2B

The aim of Experiment 2B, conducted soon after Experiment 2A, was to expand the results of this experiment by providing a new demonstration of the Rodríguez et al. (2010) protocol. Thus, the experiment offers a comparison between NPE (No Previous experience) males and females. The main prediction in this experiment was a gap between males and females in the shape test trial, with male rats performing better than female rats.

### Subjects and Apparatus

The subjects were naive Long Evans rats from our own colony: 9 males and 9 females, approximately three and a half months old at the beginning of the experiment. They were divided into two groups, males and females. The animals were housed, kept and maintained as in the previous experiments. The two groups were named Group NPE-Males and Group NPE-Females. The triangular shaped-pool, the black curtains, and the experimental room were also the same as in the previous experiments. The landmark, hidden platform, *P*, and the geometry of the pool were situated as shown in Figure 1B1.

### Results and Discussion

**FIGURE 5:**
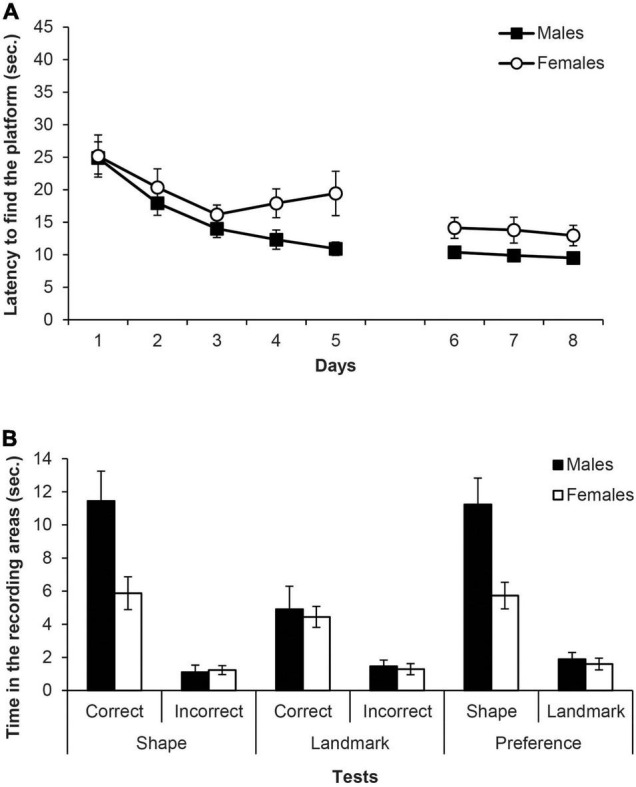
Experiment 2B. **(A)** Mean escape latencies by the subjects in Experiment 2B during the three training phases and the escape trials of the test days. Error bars denote standard error of the mean. **(B)** Mean time spent by the subjects in Experiment 2B in the two recording areas during the main test trials: Shape test and Landmark test (recording areas: correct and incorrect, C and I, respectively), and Preference test (recording areas: shape and landmark, S and L, respectively). Error bars denote standard error of means.

Figure 5A shows the mean escape latencies of male and female rats during all the experiment (days 1–8). Two mixed ANOVAs were carried out on the latencies with the within-subjects factor of Days and the between-groups factor of Sex (males, females), one for the escapes of the training phase with the triangular pool and the landmark (days 1–5) and another for the escapes of the test phase (days 6–8). The mixed ANOVA of the training phase (days 1–5) did not meet the assumption of sphericity, χ^2^(9) = 32.39, *p* < 0.001, therefore the Greenhouse-Geisser estimate of sphericity (ε = 0.62) was used to correct the degrees of freedom of the within-subject factors. The ANOVA showed that Days was the only significant factor, *F*(2.46,39.36) = 10.85, *p* < 0.001, η^2^_p_ = 0.40, and rats found the platform faster as the days went by. No other main effect or interaction was significant (*F*s ≤ 2.97). The ANOVA of the escapes on the test phase (days 6–8) also did not meet the assumption of sphericity, χ^2^(2) = 8.69, *p* = 0.013, therefore the Greenhouse-Geisser correction was applied (ε = 0.70). The ANOVA revealed that only the main factor Sex was significant, *F*(1,16) = 6.02, *p* = 0.026, η^2^_p_ = 0.27, and males escape latencies were lower than those of females. No other main effect or interaction reached significance (*F*s ≤ 0.50).

Figure 5B (left and middle) shows the time spent in the two recording areas on each learning test (i.e., shape and landmark). The mixed ANOVA of the two learning tests including the factors Area (correct, incorrect), Test (shape, landmark), and Sex revealed that the main factors Test and Area were significant, *F*(1,16) = 19.30, *p* < 0.001, η^2^_p_ = 0.55 and *F*(1,16) = 49.81, *p* < 0.001, η^2^_p_ = 0.76, respectively. Furthermore, the interactions Test × Area and Test × Sex were also significant, *F*(1,16) = 10.52, *p* = 0.005, η^2^_p_ = 0.40 and *F*(1,16) = 7.79, *p* = 0.013, η^2^_p_ = 0.33, respectively. The interaction Test × Area × Sex was close to significance, *F*(1,16) = 4.37, *p* = 0.053, and no other factor or interaction was significant (*F*s ≤ 3.97). Simple effect analyses of the interaction Test × Area found that, both in the shape and landmark tests, rats spent more time in the correct area than in the incorrect one, *F*(1,16) = 41.17, *p* < 0.001, η^2^_p_ = 0.72 and *F*(1,16) = 17, *p* = 0.001, η^2^_p_ = 0.52, respectively. Moreover, rats spent more time in the correct area of the shape test than in the correct area of the landmark test, *F*(1,16) = 14.52, *p* = 0.002, η^2^_p_ = 0.48, while there was no significant difference between the time that rats spent in the incorrect area of both tests (*p* = 0.546). Simple effect analyses of the interaction Test × Sex revealed that male rats spent more time swimming in the recording areas of the shape test than female rats, *F*(1,16) = 8.31, *p* = 0.011, η^2^_p_ = 0.34, but males and females did not differ in the time spent in the recording areas of the landmark test (*p* = 0.694). Also, male rats spent more time in the recording area of the shape test than in the landmark one, *F*(1,16) = 25.81, *p* < 0.001, η^2^_p_ = 0.62, whereas that was not the case in the female rats (*p* = 0.274).

Figure 5B (right) also shows the time in the shape and landmark areas on the preference test. The mixed ANOVA including the variables Area (shape, landmark) and Sex showed that the main effects of Area and Sex were significant, *F*(1,16) = 42,74, *p* < 0.001, η^2^_p_ = 0.73 and *F*(1,16) = 12.80, *p* = 0.003, η^2^_p_ = 0.44, respectively, as well as the interaction Area × Sex, *F*(1,16) = 6.41, *p* = 0.022, η^2^_p_ = 0.29. Simple effect analyses of the interaction Area × Sex revealed that male rats spent more time than females in the shape area *F*(1,16) = 9.64, *p* = 0.007, η^2^_p_ = 0.38, whereas male and female rats did not differ in the landmark area (*p* = 0.599). Furthermore, both male and female rats spent more time swimming in the shape area than in the landmark area, *F*(1,16) = 41.12, *p* < 0.001, η^2^_p_ = 0.72 and *F*(1,16) = 8.03, *p* = 0.012, η^2^_p_ = 0.33, respectively.

In conclusion, all rats improved their performance as days went by in the training phase, and males reached the platform faster than females. In the shape test trial, as predicted, the gap between males and females that was typically observed in the past, reappeared. The results of the female rats, both in the landmark test trial and in the preference test trial, are surprising (i.e., they do not replicate the main previous results of Rodríguez et al., 2010). Although it is speculation, it is important to say that, in the last years in our laboratory there has been a change in the behaviour of females, both in the landmark test trial and in the preference test trial. These new results by female rats in the two mentioned tests, already replicated several times, coincided with an important event. Within a short time, the lab switched to housing mostly mice instead of rats, just the opposite of what had happened in the past. It is well known that rats, upon seeing or smelling a mouse, become natural killers, changing their behaviour in order to hunt the mouse and ultimately kill it (O’Boyle, 1974; Van Hemel, 1975; Albert et al., 1982; Kemble et al., 1993). It could be the case that this natural predatory behaviour is interfering with a landmark preference in the females. As for the shape test trial, if the pool-geometry has a certain level of difficulty, the result has always been the same. Male rats perform better than female rats. All the experiments presented here were done after the “invasion” of the mice.

## General Discussion

In Experiment 1, a preliminary experiment with female rats only, no differences were found between a learning procedure (Learning rats) and a Pre-exposure procedure (Preexposure rats), neither in the escape trials on days 17 and 18 nor in the test trial. This was a surprising result, as we expected a better performance in the learning rats than in the pre-exposure animals. Consequently, LPE rats (50% of which had a learning experience and the other 50%, a pre-exposure experience) and SPE animals (50% of which had a learning experience and the other 50%, a pre-exposure experience) were considered as two main groups: LPE and SPE (i.e., Long Previous Experience and Short Previous Experience, respectively). After Training 3, the shape test trial revealed that the females of Group LPR spent more time swimming in the target corner of the pool than the females of the other two groups (Group SPE and Group NPE), which did not differ. A possible implication is that a long prior experience with geometry (i.e., one with two pool shapes, Group LPE), clearly benefited the performance of female rats in the shape test following compound training, but a short prior experience (i.e., one with one pool shape only, Group SPE), could not. Then, Experiment 2A directly compared the performance of males and females (i.e., Group LPEm and Group LPEf), while extending the procedure used with the learning rats in Group LPE in Experiment 1. In total, four test trials, without the platform, were conducted in this experiment after compound acquisition (i.e., Training 3): a shape test, a landmark test, and a preference test (as in Rodríguez et al., 2010, Experiment 2), which were counterbalanced. A final test was also conducted with an incomplete pool shape to check if rats could identify the correct corner of this pool by its local properties, instead of global properties (as Pearce et al., 2004 and McGregor et al., 2006 addressed for the first time, but only with male rats).

The results of Experiment 2A revealed that males and females did not differ in the initial learning speed to find the hidden platform in the Morris pool, neither during the pre-training escape trials, in the circular pool in the absence of the landmark, nor in the subsequent training phases with the hidden platform located in a certain corner of the kite-shaped pool (Training 1), of the rectangular-shaped pool (Training 2), and of the triangular-shaped pool–in the last case close to the landmark (Training 3). This suggests that females do not spend more time exploring the pool than males, but that both males and females seem to swim directly to the platform (see Forcano et al., 2009 and Chamizo et al., 2020 for similar results). The most relevant result of Experiment 2A is the shape test trial, without the platform (i.e., in the triangular pool and no landmark). As predicted, the gap between adult males and females that was typically observed in the past in this test when using the present protocol (Rodríguez et al., 2010, 2013; Keeley et al., 2013; Torres et al., 2014; Chamizo et al., 2016) had disappeared. Therefore, a long prior experience with geometry benefited the learning of the geometry cue of female rats following compound training. Both sexes also performed similarly in the landmark test; and in the preference test, males and females spent more time swimming in the target shape corner of the pool than in the recording area of the pool that was close to the landmark. The implication is that following compound acquisition (i.e., Training 3), all rats had learned the same, less about the landmark cue than about the geometry cue, and both males and females preferred the geometry cue to the landmark cue. Therefore, the geometry of the pool was dominant over the rats’ behaviour even though there was another cue during compound training, the landmark, signalling the correct corner which was an equally good predictor of the hidden platform.

The present results, at least in adult female rats, are not easy to explain by appealing to the concept of core or innate knowledge (Spelke and Kinzler, 2007), an explanation which, however, could be possible with young, prepubertal female rats, which behave like male rats in the preference test of the present protocol (Rodríguez et al., 2013, Experiments 2a and 2b). The results of Experiments 2a and 2b revealed that, quite contrary to what occurs in adult rats, 30-day-old females, like males of the same age, preferred the geometry cue to the landmark cue. These results were confirmed and expanded in Experiments 3–5. The age effects found in the study by Rodríguez et al. (2013) in part replicates an earlier age effect reported by Kanit et al. (2000). In this study (Kanit et al., 2000) it was also observed that young female rats behaved like male rats, but in a different way than adult females, in their search for the hidden platform in a Morris pool. Rodríguez et al. (2013) wondered how it could be explained that the behaviour of female rats changed as they aged. It was speculated that the most likely answer, based on the observation that ovariectomised females show a behaviour that is similar to that of younger rats and not like adult rats, is that the hormonal changes associated with the onset of puberty is responsible for the change in the behaviour of adult females. The fact that sex differences in spatial tasks are found only after puberty, although young female rats behave similarly to male rats, is compatible with the proposal, claimed for human subjects, that when dealing with spatial cognition sex differences are observed only from the age of approximately 10–12 years (for reviews, see Linn and Peterson, 1985; Voyer et al., 1995).

Interestingly, in the study by Chamizo et al. (2016), addressing the effects of early stimulation, four groups of male and female rats were trained while using the present protocol (Rodríguez et al., 2010, Experiment 2). This study clearly showed that the combination of an enriched environmental experience and voluntary wheel running was insufficient to counteract or modulate the effects caused by the hormonal changes that are associated with the onset of puberty in female rats (Kanit et al., 2000; Rodríguez et al., 2013). The environmental enrichment (EE) protocol that was employed could not change adult females’ preference for the landmark cue. And when the two cues were tested individually (i.e., the learning test trials), although a clear beneficial effect was found in all EE rats, males in the two groups (i.e., EE and control) performed better on the shape test than on the landmark test, and males in both groups also spent more time swimming while searching for the hidden platform in the target area on the shape test than did females, while males and females did not differ on the landmark test. Therefore, as in Rodríguez et al. (2010), a clear male advantage on shape learning was found (for a review see Chamizo and Rodrigo, 2019).

The main results of the present Experiment 2A, those of the shape test trial (in the absence of the landmark), where males and females did not differ (i.e., the sex gap had disappeared) can be explained by selective attention (Mackintosh, 1975; for a review of selective attention theories see Zentall and Riley, 2000). The attentional theory by Mackintosh (1975) states that changes in the associability of, or attention to, particular stimuli, depend on their relative predictive value. According to this theory, attention is selective. This implies that if the attention to certain stimuli is increased, this will necessarily have as a consequence that the attention paid to other stimuli will decrease. A study of landmark learning by Trobalon et al. (2003), with rats in a radial maze, supports this theory. The rats were always trained in two discriminations and the four experiments of this study compared intradimensional (ID) and extradimensional (ED) changes. The results revealed that the rats learned the second discrimination faster if the relevant stimuli were from the same dimension in the two discriminations (i.e., an intradimensional or ID shift), but they were slower if the relevant stimuli in the two discriminations were from different dimensions (i.e., an extradimensional or ED shift). Specifically, Trobalon et al. (2003) found that when landmark learning, rats trained on a spatial discrimination do not learn to attend to all spatial landmarks but only to those that serve to differentiate S+ and S- (i.e., S+, signals the availability of reinforcement; and S–, the absence of reinforcement). The study by Trobalon et al. (2003) constitutes an example of selective attention in the spatial domain. A similar selective attention could be expected when successive geometry problems are presented to the rats in the present experiments (i.e., on Training 1 and Training 2 with LPE animals) and the location of the platform is discovered only by reference to a target corner–being geometry the relevant dimension. This prior experience with different pool-shapes forced selective attention in the rats, without other stimuli that could interfere, and seems responsible for the unusual learning of the females about the geometry cue on Training 3, which was tested with the triangular pool and no landmark. Interestingly, this result could imply that the previous experience with geometry has benefited adult female rats more than adult male rats (for similar results in humans and visuospatial tasks, see the review by Castro-Alonso and Jansen, 2019). Has a bias, possibly acquired during the evolutionary history of the adult females of this species (*Rattus norvegicus*), been counteracted in the present experiments? Could the present results end up supporting the idea of innate or core knowledge to capture the geometry of the environment, as proposed by Spelke and Kinzler (2007)? Further research will have to answer these questions. Experiments with fish support the idea that the encoding of environmental geometry is innate and independent from early experience (Brown et al., 2007; Sovrano and Chiandetti, 2017). Anyway, further research, also across species, will have to answer these questions.

A final aim of Experiment 2A was to elucidate, both in male and female rats, the importance of local properties and global properties following shape learning, as Pearce et al. (2004–see also Tommasi and Polli, 2004; Esber et al., 2005; McGregor et al., 2006) have already done, but with male rats only. In the study by McGregor et al. (2006), male rats were trained to find a hidden platform in a specific corner of a pentagon-shaped pool. Subsequent test trials in a rectangular-shaped pool revealed that the rats searched for the platform preferably in the corners that were congruent with the previously correct corner in the pentagon-shaped pool. Moreover, when the rats were released into the rectangular arena it was observed that they headed directly for a correct corner. Further test trials confirmed these results. The implication is that the males’ navigation in an environment that had a specific shape was based on local and not global cues–a result which is the opposite to that predicted by Cheng and Gallistel (2005). These authors suggest that the navigation of rats is based on global cues (i.e., on the main axis of the shape of a certain apparatus or environment). Cheng (1986; see also 2005) had claimed that such a type of learning is represented in a specific module, a geometric one. As in the study by McGregor et al. (2006), the final test trial in Experiment 2 revealed that both, male and female rats could identify the correct corner of the specific pool-shape (i.e., the kite-shaped pool) on the basis of its spatial relationship with local cues, instead of a global representation of the pool shape, thus supporting their results.

Finally, Experiment 2B provides a new demonstration of the Rodríguez et al. (2010) protocol, in an attempt to compensate for the lack of control groups (i.e., male and female rats without previous experience) in Experiment 2A. The main prediction in this experiment was a difference between males and females in the shape test trial, with male rats performing better than female rats. As was the case. Experiment 2B also shows possible problems (specifically, in the landmark test trial and in the preference test trial), that could be due to the fact of bringing together different species in the same laboratory. A subject very little studied (as an exception see Arndt et al., 2000).

The present study shows for the first time that a long previous experience with different pool shapes has eliminated the sex gap repeatedly found in previous studies with the present protocol, firstly found by Rodríguez et al. (2010). Although the reasons for this are not fully clear, we are inclined to conclude that our procedure has counteracted an existing attentional bias in our female rats. The present results could have important implications for women as, in general, they seem to have less spatial abilities than men (for a whole thematic series addressed to answer responses of why spatial abilities are crucial in education, learning, and everyday activities see Ishikawa and Newcombe, 2021). Spatial abilities correlate significantly with STEM (acronym for science, technology, engineering, and mathematics) disciplines, as well as with the choice of future careers and occupations; and most importantly, they can be learned! (Uttal et al., 2013; Sorby et al., 2018). It has been shown (Haier et al., 2009) that even simple spatial activities, such as a frequent use of the game Tetris (which consists of matching pieces that fall in circles from the top of a screen, to complete a wall without leaving gaps), can favourably alter the plasticity of the brain of girls. The practice of sport can also have important effects on spatial abilities (Jansen et al., 2018; Meneghetti et al., 2021). Good spatial skills are also important to healthy aging (Sodums and Bohbot, 2020) and to prevent Alzheimer’s disease (Li and Singh, 2014). For all these reasons, we believe that spatial abilities should be taken into account in the design of clinical and educational procedures, which could be beneficial for the population in general and for the female spectrum in particular.

## Data Availability Statement

The raw data supporting the conclusions of this article will be made available by the authors, without undue reservation.

## Ethics Statement

The animal study was reviewed and approved by the Ethics Committee of the Universitat de Barcelona. In the two experiments all animal treatment and care abided by the ethical principles of the aforementioned university regarding the care and use of animals for scientific purposes, as well as by the corresponding principles of the European Community (EEC Council Directive 86/609/EEC).

## Author Contributions

AA-L and VR-N ran the experiments. EG conducted the statistics and graphic designs. VC designed the experiments and wrote the manuscript. All authors contributed to the article and approved the submitted version.

## Conflict of Interest

The authors declare that the research was conducted in the absence of any commercial or financial relationships that could be construed as a potential conflict of interest.

## Publisher’s Note

All claims expressed in this article are solely those of the authors and do not necessarily represent those of their affiliated organizations, or those of the publisher, the editors and the reviewers. Any product that may be evaluated in this article, or claim that may be made by its manufacturer, is not guaranteed or endorsed by the publisher.
